# Supplementary material for: Electroacupuncture versus sham electroacupuncture in treating low anterior resection syndrome after rectal cancer surgery: Study protocol for a randomized controlled trial
Source: PLoS One. 2025 Jul 30;20(7):e0329035. doi: 10.1371/journal.pone.0329035 (PMC12310008; doi:10.1371/journal.pone.0329035)
Supplement: S2 File — (DOCX) [file pone.0329035.s002.docx]

**Electroacupuncture versus sham electroacupuncture in the treatment of low anterior resection syndrome after resection for rectal cancer: a multi-center randomized controlled trial**

**Project leader：Cun-Zhi Liu**

**Undertaker: Beijing University of Chinese Medicine**

**Cooperative Units:**

**Beijing Friendship Hospital Affiliated to Capital Medical University**

**Cancer Hospital of Chinese Academy of Medical Sciences**

**the First Affiliated Hospital of Zhengzhou University**

**1. Research content**

Study subjects: patients with Low anterior resection syndrome

Intervention: electroacupuncture

Effect indicators: improvement in Low anterior resection syndrome score

Study type: Randomized controlled trial

**2. Protocol**

**2.1 Participants**

2.1.1 Western diagnostic criteria

low anterior resection syndrome (LARS) is defined as a patient who has at least one of the following eight symptoms and causes at least one of the following eight consequences after total mesorectal excision or low anterior resection for rectal cancer: Eight symptoms (variable/unpredictable bowel function, intermittent bowel movements, increased bowel frequency, pain during repeated bowel movements, difficulty emptying, urgency to bowel movements, fecal incontinence, defecation) and eight consequences (toilet dependence, excessive concern with bowel function, dissatisfaction with bowel function, need to use coping strategies to manage bowel function, psychological and emotional health, social and daily activities, intimacy Relationships, influencing roles, commitments and responsibilities).

2.1.2 TCM syndrome diagnostic criteria

N/A

2.1.3 Inclusion criteria

① Age between 18 and 75 years of any gender;

② Diagnosis of LARS confirmed at least one month after rectal resection or stoma closure (LARS score ≥ 21).

③ Provide written informed consent.

2.1.4 Exclusion criteria

① History of other types of colorectal cancer surgery or resection of other intestinal segments, including Hartmann’s procedure, abdominopelvic resection, transanal endoscopic microsurgical resection, or sigmoid colon resection;

② History of other pelvic surgeries for non-neoplastic conditions;

③ Preoperative fecal incontinence;

④ History of mental disorders, alcohol abuse, or drug abuse;

⑤ Diagnosis of inflammatory bowel disease or irritable bowel syndrome;

⑥ Received acupuncture treatment within the past one months;

⑦ Presence of implantable medical devices, such as a cardiac pacemaker;

⑧ Participated in another clinical study;

2.1.5 Criteria for Withdrawal (Dropout)

① Participants who have signed informed consent, passed eligibility screening, and entered the randomized trial but subsequently discontinue the intervention or fail to complete the observation period as outlined in the protocol will be classified as dropout cases;

② In the event of a participant’s withdrawal, investigators should make every effort to contact the individual, document the reasons for withdrawal, and complete as many assessments as possible. All relevant trial records for dropout participants must be properly retained for archiving and for inclusion in full analysis set (FAS) statistical processing. Participants who withdraw will not be replaced.

2.1.6 Suspension criteria

A participant will be withdrawn from the study under any of the following conditions:

① The occurrence of serious adverse events (SAEs), severe complications, or the need for reoperation, where the investigator determines that continued participation is not in the patient’s best interest;

② The development of other medical conditions that interfere with study assessments or make completion of acupuncture treatment infeasible, as judged by the investigator; such participants will be considered treatment failures;

③ Significant protocol deviations during trial implementation that compromise the evaluation of acupuncture efficacy;

④ The participant voluntarily requests to withdraw from the clinical trial during the study period by notifying the attending physician.

**2.2 Sample size**

Based on previous study, we speculated that the LARS score would be decreased by 6.8 points in the electroacupuncture group and 4.1 points in the sham-electroacupuncture group, with a standard deviation of 5. Considering a 20% dropout rate, it is estimated that a total of 136 subjects will need to be recruited.

**2.3 Randomization and Blinding Procedures**

A stratified block randomization method will be employed, with stratification based on study centers. Eligible participants will be randomly assigned in a 1:1 ratio to either the electroacupuncture (EA) group or the sham acupuncture (SA) group. The randomization sequence will be generated using SAS software by an independent statistician who is not involved in any aspect of the trial. Randomization numbers will be managed and safeguarded by a designated randomization coordinator, who will not participate in the intervention, outcome assessment, or statistical analysis.

For each eligible participant enrolled, the screening personnel will obtain the corresponding randomization number by phone from the randomization coordinator. Throughout the trial, the randomization sequence will remain concealed from all other study personnel to ensure allocation concealment.

Blinding will not be applied to the acupuncturists due to the nature of the intervention. However, patients, outcome assessors, and statistical analysts will remain blinded to group allocation to minimize potential bias.

**2.4 treatment protocol**

Participants will be randomly assigned in a 1:1 ratio to either the electroacupuncture (EA) group or the sham acupuncture (SA) group. Acupuncture interventions will be administered by licensed Traditional Chinese Medicine (TCM) acupuncturists at each study center. All practitioners are required to hold a valid TCM physician license issued by the People’s Republic of China and possess at least three years of clinical experience.

A standardized acupuncture protocol will be employed in the EA group, with two alternating sets of primary acupoints:

Set 1: Zhongliao (BL33), Ciliao (BL32), Sanyinjiao (SP6), and Taixi (KI3)

Set 2: Tianshu (ST25), Shuidao (ST28), Zusanli (ST36), and Yinlingquan (SP9)

Only one set of acupoints will be used per treatment session, with the two sets alternated throughout the intervention period. Detailed acupoint locations are provided in Table 1.

Sterile, single-use Hwato-brand acupuncture needles (0.40 mm × 30 mm or 0.25 mm × 30 mm) will be used for all procedures. Prior to needling, both the acupoint sites and the practitioner’s hands will be disinfected thoroughly with 75% alcohol. Manual needle insertion will follow the balanced reinforcing-reducing technique through bidirectional rotation for 30 seconds to elicit the “deqi” sensation. After achieving deqi:

In Set 1, electroacupuncture stimulation will be applied to bilateral BL32 and paired between SP6 and KI3.

In Set 2, stimulation will be applied to bilateral ST25 and paired between ST36.

The intensity of electrical stimulation will be adjusted to the maximum tolerable level that produces slight tremor at the needle handle. A continuous wave at 10 Hz will be used, and all needles will be retained in situ for 30 minutes.

**Table 1. Location of acupoints in EA group.**

| **Acupoints** | **Locations** |
| --- | --- |
| Zhongliao (BL33) | In the sacral region, opposite the third sacral posterior foramen |
| Ciliao (BL32) | In the sacral region, the second sacral posterior foramen |
| Sanyinjiao (SP6) | On the medial of the lower leg, 3 cun^a^ above the tip of the medial malleolus, posterior to the medial margin of the tibia |
| Taixi (KI3) | On the medial side of the foot, in the depression between the medial tip of the ankle and the Achilles tendon |
| Tianshu (ST25) | On the abdomen, level with the umbilicus, 2 cun lateral to the anterior midline |
| Shuidao (ST28) | On the abdomen, 3 cun below the umbilicus, 2 cun lateral to the anterior midline |
| Zusanli (ST36) | On the anterolateral side of the lower leg, 3 cun below the Dubi (ST35) point, one finger-width (middle finger) lateral to the anterior crest of the tibia |
| Yinlingquan (SP9) | On the medial side of the lower leg, in the depression between the inferior border of the medial condyle of the tibia and the medial border of the tibia |

^a^1 cun (≈20 mm) is defined as the width of the interphalangeal joint of patient’s thumb

Participants in the sham acupuncture group will receive superficial needling without manual stimulation and without the elicitation of the deqi sensation. Two sets of non-acupoints (Non-acupoints 1–4 and Non-acupoints 5–8) will be used alternately in each session. Sham electroacupuncture will be applied by attaching electrodes to bilateral Non-acupoint 4 and Non-acupoint 8.Although the electrode wires will be connected to the electroacupuncture device, no electrical current will be delivered. Specifically, the red wires used for stimulation will be disconnected internally to ensure no active current passes through. All needles will remain in place for 30 minutes.

**Table 2. Location of acupoints in SA group.**

| **Acupoints** | **Locations** |
| --- | --- |
| non-acupoint 1 | In the sacral region, 1 cun^a^ lateral to the Zhongliao (BL33) point |
| non-acupoint 2 | In the sacral region, 1 cun lateral to the Ciliao (BL32) point |
| non-acupoint 3 | On the lower limb, 2 cun above the medial malleolus, the medial side of the tibia is median, between the Liver and Spleen meridians |
| non-acupoint 4 | On the lower limb, at the midpoint of the line connecting Qiuxu (GB40) and Jiexi (ST41) points |
| non-acupoint 5 | On the abdomen, 2 cun below the umbilicus and 1 cun apart from the midline |
| non-acupoint 6 | On the abdomen, 2 cun above the anterior superior iliac spine |
| non-acupoint 7 | On the lower limb, on the lateral side of the lower leg, 3 cun below the Yanglingquan (GB34) point, between the Gallbladder and Bladder meridians |
| non-acupoint 8 | On the lower limb, on the medial side of the lower leg, 1 cun lateral to the Chengjin (BL56) point, between the Bladder and Stomach meridians |

^a^1 cun (≈20 mm) is defined as the width of the interphalangeal joint of patient’s thumb

Participants in both groups will receive acupuncture three times per week during the first four weeks (Weeks 1–4), and twice per week during the subsequent four weeks (Weeks 5–8), for a total of 20 sessions over an 8-week treatment period. No additional therapies targeting low anterior resection syndrome (LARS) will be permitted during the study period.

In cases of severe fecal incontinence, participants may be allowed to take loperamide hydrochloride capsules (Imodium) as needed for symptom relief.

**Combined Medication Regulations:**

The related medication was recorded

**2.5 Outcome Measures**

**Primary outcome**

Change in LARS score from baseline at week 8.

The LARS score is a validated self-rating scale to assess bowel function after low rectal resection and consists of five items: fecal incontinence, urinary incontinence, bowel frequency, bowel aggregation, and urgency, with scores ranging from 0 to 42. The severity of bowel dysfunction can be categorized as: no LARS (0-20), mild LARS (21-29), or severe LARS (30-42).

**Secondary outcome**

① Changes in LARS Score from Baseline at Week 2, 4, 6, 12 and 24.

② Proportion of Patients with Improvement in Bowel Dysfunction

At weeks 2, 4, 6, 8, 12, and 24, the number of patients improving from severe LARS to mild LARS, from mild LARS to no LARS and from severe LARS to no LARS will be determined according to LARS score.

③ Subjective distress of intestinal symptoms

We will assess subjective distress of intestinal symptoms by the Numerical Rating Scale (NRS) at Week 4, 8, 12 and 24, where 0 indicates no distress, and 10 represents the worst imaginable distress.

④ EORTC-QLQ-C30 Quality of Life Questionnaire

The core quality of life questionnaire developed by the European Organization for Research and Treatment of Cancer (EORTC-QLQ-C30), comprising 30 items. It includes 5 functions, 3 symptoms, 1 global health status, and 6 single-item measures. Higher scores on the functional scales indicate better quality of life, whereas higher scores on the symptom scales indicate worse quality of life. Patients were evaluated at weeks 4, 8, 12, and 24.

⑤ Bristol Stool Diary

During the week 0, 4, 8, 12 and 24, we will record bowel movement frequency, stool consistency and urgency over the past week.

⑥ Fecal Incontinence Quality of Life Scale (FIQL)

We will evaluate the quality of life in patients with fecal incontinence, covering four aspects: lifestyle changes, coping/behavior limitations, depression/self-perception, and social embarrassment at week 0, 4, 8, 12 and 24.

⑦ Diarrhea Wexner Score:

we will evaluate at week 0, 4, 8, 12 and 24.

⑧ Credibility and Expectancy Assessment:

The credibility of the intervention and participants’ treatment expectations will be evaluated immediately following the first treatment session.

⑨ Blinding Assessment:

Blinding effectiveness will also be assessed at the end of the first treatment session.

**2.6 Efficacy Evaluation Criteria**

The primary indicator of treatment efficacy will be the change in LARS score from baseline.

**2.7 Adverse Events**

Any adverse reactions occurring during acupuncture treatment will be documented, including but not limited to bleeding, subcutaneous hematoma, numbness, and post-needling sensations such as soreness or distension. In cases of needle fainting (vasovagal response), acupuncture should be discontinued immediately, all needles withdrawn, and the patient placed in a supine position with warmth provided.

Minor subcutaneous bleeding resulting in small ecchymosis typically requires no intervention and will resolve spontaneously. If the affected area shows significant swelling, pain, or a large ecchymotic patch, cold compresses will be applied to reduce bleeding. For mild soreness or distension after treatment, local manual massage is recommended to relieve symptoms. In more severe cases, additional therapies may be used under medical supervision.

All adverse events will be managed symptomatically. If the acupuncturist is unable to resolve the issue, consultation with an appropriate medical specialist will be arranged. Any costs arising from adverse events related to the study intervention will be handled in accordance with applicable laws and regulations.

Assessment time point: Throughout the treatment period.

**2.8 Data Entry and Statistical Analysis**

A modified intention-to-treat (mITT) approach will be applied. All randomized participants who meet inclusion criteria will be included in the statistical analysis. Continuous variables will be expressed as mean ± standard deviation (M ± SD) or as median with interquartile range, depending on distribution. Categorical variables will be presented as frequencies, proportions, or percentages.

Baseline demographic and clinical characteristics will be compared using analysis of variance (ANOVA) or chi-square (χ²) tests. Between-group differences in outcomes will be analyzed using t-tests, rank-sum tests, ANOVA, or χ² tests, as appropriate. For repeated measures outcomes, repeated-measures ANOVA will be employed.

All statistical tests will be two-sided, with a significance level of α = 0.05. A p-value < 0.05 will be considered statistically significant. Statistical analyses will be conducted using SPSS software.

**2.9 Sample Collection**

N/A

**2.10 Quality Control**

① Participant recruitment will strictly follow the predefined diagnostic, inclusion, and exclusion criteria to ensure appropriate subject selection.

② Robust measures will be implemented to maintain allocation concealment. Randomization numbers will be managed by an independent randomization coordinator not involved in the trial. A randomization number will be issued only after the subject’s eligibility has been confirmed and the relevant information has been properly documented. This process ensures allocation concealment and prevents premature disclosure or manipulation of random sequences.

③ Standard Operating Procedures (SOPs) will be developed for all stages of the trial following expert consultation. These procedures will standardize trial conduct across sites and provide a clear reference in cases of disagreement.

④ All research staff must undergo centralized training, which includes the study’s objectives, protocol requirements, diagnostic and treatment standards, randomization procedures, acupuncture techniques, and the use of evaluation tools. Training duration will vary by role, and investigators assigned to the same role at different centers must pass a consistency assessment before participating. Video records of these assessments will be archived for quality assurance.

⑤ A trial management team will be established, led by the principal investigator. Center-specific monitors will be appointed to oversee trial conduct and ensure quality control at each site.

⑥ A unified, preprinted case report form (CRF) will be used across all sites. Each CRF will be numbered and its assignment recorded. Investigators must complete CRFs objectively, accurately, and in accordance with the trial protocol. Any issues or deviations encountered during the study must be truthfully recorded.

⑦ To maintain the integrity of the blinding process, principal investigators and data analysts will remain blinded to group assignments. Study functions—including participant recruitment, treatment administration, data collection, entry, and statistical analysis—will be performed independently by different personnel.

⑧ Patient compliance will be promoted through comprehensive education on the study’s purpose and significance. Written informed consent will be obtained from all participants. Acupuncture treatment costs will be fully covered by the study’s funding.
