# Supplementary material for: Electroacupuncture versus sham electroacupuncture in treating low anterior resection syndrome after rectal cancer surgery: Study protocol for a randomized controlled trial
Source: PLoS One. 2025 Jul 30;20(7):e0329035. doi: 10.1371/journal.pone.0329035 (PMC12310008; doi:10.1371/journal.pone.0329035)
Supplement: S3 File — (DOCX) [file pone.0329035.s003.docx]

**Model consent form**

**Informed Consent Form • Informed Notice Page (Translated Version)**

Dear Madam/Sir,

You are invited to participate in clinical study titled “Electroacupuncture versus Sham Electroacupuncture in the Treatment of Low Anterior Resection Syndrome (LARS) after Resection for Rectal Cancer: A Multicenter Randomized Controlled Trial”. The purpose of this study is to evaluate the effectiveness and safety of electroacupuncture in the treatment of LARS.

Before you decide whether to participate in this study, please carefully read the following information. It will help you understand the purpose of the study, its procedures and duration, as well as the potential benefits, risks, and discomforts associated with participation. If you wish, you may discuss it with your relatives and friends or ask your doctor for an explanation to help you decide.

**Introduction**

1. We will conduct the " Electroacupuncture versus Sham Electroacupuncture in the Treatment of Low Anterior Resection Syndrome (LARS) after Resection for Rectal Cancer: A Randomized Controlled Trial " The principal investigator of this project is Professor Cunzhi, Liu, Dean of the School of Acupuncture-Moxibustion and Tuina, Beijing University of Chinese Medicine.

2. This is a clinical trial.

3. Some patients undergoing total mesorectal excision, low anterior resection (LAR), and other procedures are defined as LARS when they experience at least one of the following eight symptoms and cause at least one of the following eight consequences: Eight symptoms (variable/unpredictable bowel function, intermittent bowel movements, increased bowel frequency, pain during repeated bowel movements, difficulty emptying, urgency to bowel movements, fecal incontinence, defecation) and eight consequences (toilet dependence, excessive concern with bowel function, dissatisfaction with bowel function, need to use coping strategies to manage bowel function, psychological and emotional health, social and daily activities, intimacy Relationships, influencing roles, commitments and responsibilities). Its high incidence and disability rates not only impose a heavy economic burden on patients but also significantly affect their quality of life. Although drug therapy for LARS has shown promising short-term efficacy, many patients experience varying degrees of adverse reactions. As a simple and efficient traditional Chinese medicine therapy, acupuncture and moxibustion has the effect of regulating pelvic floor neuromuscular function and promoting surgical recovery. It has high safety and few side effects and is widely used and welcomed by many patients.

4. This study will include 136 patients with LARS. The study will last for approximately 24 weeks. Before enrollment in the study, the researcher will refer to the inclusion and exclusion criteria, inquire about your relevant symptoms, and inform you of the details of the study, addressing all related issues. If you enter the trial, you will have an equal chance of being assigned to either the electroacupuncture group or the micro-electroacupuncture group and receive the corresponding treatment. You will receive treatment three times a week for weeks 1-4 and twice a week for weeks 5-8. Regardless of the group you are assigned to, visits and relevant scale assessments will be conducted during treatment and follow-up. These assessments will include evaluation of the impact of electroacupuncture on intestinal function after low rectal resection and function in patients using questioners such as the LARS score, Numerical Rating Scale, EORTC-QLQ-C30 Quality of Life Scale and observation of safety of electroacupuncture through assessment of adverse events. Throughout the trial, you may receive Loperamide Hydrochloride Capsule as a temporary oral analgesic when experiencing severe fecal incontinence. Please keep all packaging and remaining medication, as the researcher will collect these materials for documentation at the end of the trial.

5. Inclusion criteria

You will be eligible if you: aged 18-75, any gender; meet LARS diagnosis and the LARS score ≥21 after rectal resection or 1 month after stoma closure; are willing to sign the informed consent.

6. Exclusion criteria
You will be ineligible if meet the following points: Other types of colorectal cancer surgery or resection of other intestinal segments, such as Hartmann surgery, abdominopelvic resection, transanal endoscopic microsurgical resection, or sigmoid colon resection; received other pelvic surgery for non-tumor reasons; preoperative fecal incontinence; severe acute or chronic organic or neuropsychiatric disorder; inflammatory bowel disease or irritable bowel syndrome; received acupuncture for the last 1 months; pacemakers or other implantable medical devices; participated in other clinical studies.

7. Withdrawal Criteria:

You can withdraw from the study if:

(1) Inadequate efficacy is observed;

(2) Intolerance to adverse reactions occurs;

(3) You wish to pursue alternative treatment methods;

(4) Or withdraw from the trial without providing any reason.

8. Termination Criteria:

Your intervention may be terminated if:

(1) Significant abnormalities in vital organ function occur;

(2) Drug allergic reactions are observed;

(3) Poor compliance is noted;

(4) Deterioration of the condition or occurrence of serious adverse reactions necessitates cessation of trial treatment;

9. Post Exclusion Criteria:

You may be excluded from the study if:

(1) You violate the inclusion and exclusion criteria to enter the trial;

(2) You use concomitant medication not specified in the protocol;

(3) You are assigned to the wrong treatment group;

(4) Extremely poor treatment compliance.

10. Study Suspension/Termination Criteria:

The study may be suspended or terminated if:

(1) Effective assurance of subject safety cannot be maintained;

(2) The sponsor fails to submit required safety updates during the research and development period;

(3) The sponsor fails to promptly address and report suspected and unexpected serious adverse reactions;

(4) Evidence indicates that the study intervention is ineffective;

(5) Falsification occurs during the course of the clinical trial;

(6) Other violations of the quality management standards for clinical trials occur. When a significant, unexpected serious adverse reaction occurs during the clinical trial, or when evidence indicates serious quality issues with the clinical trial, the sponsor should immediately suspend the clinical trial. Relevant departments may, according to their responsibilities, order adjustments to the clinical trial protocol, suspend, or terminate the clinical trial.

**Explanation of Potential Benefits of Participating in the Study**

Benefits to the Social Community:

We hope that the information obtained from your participation in this study will benefit patients with similar conditions in the future.

Benefits to the Participants Themselves:

Research indicates that electroacupuncture can improve the quality of life and symptoms associated with LARS. In this study, the frequency of electroacupuncture treatment for lars was changed from 3 times a week to 2 times a week, which may improve symptoms such as the frequent and urgent bowel movements, thus enhancing your quality of life.

**Explanation of Potential Discomforts and Risks to Participants**

Discomfort caused using investigational drugs or devices:

Adverse reactions may occur during the trial. Previous clinical studies have shown that electroacupuncture has not resulted in serious adverse reactions. Common adverse events associated with electroacupuncture may include mild events such as bruising, fainting, needle retention, and soreness after treatment. If any of these occur, treatment will be paused until the adverse reaction subsides, and a decision will be made whether to continue treatment.

Risks associated with participation in the trial itself:

When considering whether to participate in this study, please carefully consider the potential impact of treatment and follow-up on your daily work, family life, etc. Consider the time and transportation issues for each treatment session and follow-up visit. If you have any questions about the content of the trial, feel free to consult us. For your safety and to ensure the validity of the study results, you will not be allowed to participate in any other clinical trials related to acupuncture, drugs, and medical devices during the study period.

**Alternatives to Participation in the Study**

You may choose not to participate in this study, which will not have any adverse effects on your access to conventional treatment. Currently, conventional treatment methods for your health condition include Re-operation, loperamide capsule to relieve diarrhea symptoms and traditional Chinese medicine may be an option.

**Instructions on Concomitant Medications and Restrictive Treatments**

During the trial, please refrain from undergoing any other treatments that may affect the symptoms of LARS, including drug therapy and other physical therapies (such as massage, hydrotherapy, etc.). If you experience severe fecal incontinence during the trial, you may request Loperamide Hydrochloride Capsules (Imodium, Xi 'an Janssen Pharmaceutical Co., LTD) 4mg as emergency medication.

**Related Expenses of Participating in the Study**

The cost of 24 sessions of electroacupuncture treatment and the as-needed use of Loperamide hydrochloride capsules will be covered by the research institution.

**Provisions for Compensation and Compensation**

When your health is harmed during your participation in this study, please inform the investigator (Liu Cunzhi, Phone number: xxxxxxxxxxx), and necessary medical measures will be taken. Compensation will be provided according to the degree of damage in accordance with relevant national laws and regulations.

**Confidentiality of the Study**

All information related to you, including your identity, medical history, condition, physical examinations, and laboratory test results, will be kept strictly confidential within the limits permitted by law. Investigators, authorized inspectors appointed by the sponsor, ethics committees, and the National Medical Products Administration are allowed to access your medical records related to this study to verify the authenticity and accuracy of the data collected in this study, without involving your personal details. Your name will not appear in any public documents or reports related to this study.

**Clarification of Participants' Rights**

Your participation in the study is entirely voluntary, and you have the right to withdraw from the study at any stage without penalty or loss of benefits, and it will not affect the treatment provided by your doctor. If you decide not to participate in this study or withdraw from the study at any time after it starts, please contact your doctor promptly.

**Handling of Participant Complaints**

If you have any complaints during the study, please contact the principal investigator (Phone number: xxxxxxxxx) or the office of the ethics committee (Phone number: xxxxxxxx).

**Informed Consent Form • Consent Signature Page**

**Participant**

I have carefully read the " Electroacupuncture versus Sham Electroacupuncture in the Treatment of Low Anterior Resection Syndrome (LARS) after Resection for Rectal Cancer: A Randomized Controlled Trial" and fully understand the purpose, content, methods, as well as the potential benefits and risks of participating in the clinical study. The researcher has provided clear explanations of relevant medical terms, and all questions I asked have been answered in a clear and understandable manner. I understand that I can refuse to join the study or withdraw from it at any time and under any circumstances, without affecting my medical treatment or rights. My participation in this study is entirely voluntary, and I have given it full consideration. I understand the therapeutic effects and potential risks that the study interventions may have on my condition, and I have obtained comprehensive and truthful information related to this study. I fully understand and support this clinical research. In the absence of any pressure and with the freedom to choose, I voluntarily participate in this clinical study and agree to cooperate with the study doctor, adhere to the prescribed medication, undergo examinations as required, and complete this clinical study. I agree that when necessary, personnel from the National Medical Products Administration, clinical research inspectors, and monitors may review my medical records and research data. I will receive a copy of the informed consent form signed and dated.

Patient (Signature): _______________ Date: ____ Year ____ Month ____ Day___

(Or Legal Guardian (Signature):______) Relationship with Patient: ____________

Phone Number: __________________

**Researcher**

I confirm that I have provided detailed explanations of the content, procedures, potential risks, and benefits of this study to the participant mentioned above. I have answered any questions raised by the patient satisfactorily, and the patient has expressed understanding and satisfaction with the responses provided.

Researcher (Signature): _____________ Date: ____ Year ____ Month ____ Day___

Phone Number: __________________
